# Application of a diagnosis-based clinical decision guide in patients with low back pain

**DOI:** 10.1186/2045-709X-19-26

**Published:** 2011-10-21

**Authors:** Donald R Murphy, Eric L Hurwitz

**Affiliations:** 1 Rhode Island Spine Center, 600 Pawtucket Avenue, Pawtucket, RI 02860 USA; 2 Department of Health Services, Policy and Practice, Alpert Medical School of Brown University, Providence, RI USA; 3 Department of Research, New York Chiropractic College, Seneca Falls, NY USA; 4 Department of Public Health Sciences, John A. Burns School of Medicine, University of Hawaii at Mānoa, Hawaii USA

**Keywords:** low back pain, diagnosis, therapeutics, practice-based research

## Abstract

**Background:**

Low back pain (LBP) is common and costly. Development of accurate and efficacious methods of diagnosis and treatment has been identified as a research priority. A diagnosis-based clinical decision guide (DBCDG; previously referred to as a diagnosis-based clinical decision rule) has been proposed which attempts to provide the clinician with a systematic, evidence-based means to apply the biopsychosocial model of care. The approach is based on three questions of diagnosis. The purpose of this study is to present the prevalence of findings using the DBCDG in consecutive patients with LBP.

**Methods:**

Demographic, diagnostic and baseline outcome measure data were gathered on a cohort of LBP patients examined by one of three examiners trained in the application of the DBCDG.

**Results:**

Data were gathered on 264 patients. Signs of visceral disease or potentially serious illness were found in 2.7%. Centralization signs were found in 41%, lumbar and sacroiliac segmental signs in 23% and 27%, respectively and radicular signs were found in 24%. Clinically relevant myofascial signs were diagnosed in 10%. Dynamic instability was diagnosed in 63%, fear beliefs in 40%, central pain hypersensitivity in 5%, passive coping in 3% and depression in 3%.

**Conclusion:**

The DBCDG can be applied in a busy private practice environment. Further studies are needed to investigate clinically relevant means to identify central pain hypersensitivity, poor coping and depression, correlations and patterns among the diagnostic components of the DBCDG as well as inter-examiner reliability and efficacy of treatment based on the DBCDG.

## Background

Low back pain (LBP) affects approximately 80% of adults at some time in life [1] and occurs in all ages [2,3]. Despite billions being spent on various diagnostic and treatment approaches, the prevalence and disability related to LBP has continued to increase [4]. There has been a recent movement toward comparative effectiveness research [5], i.e., research that determines which treatment approaches are most effective for a given patient population. In addition, there is increased recognition of the importance of practice-based research which generates data in a "real world" environment as a tool for conducting comparative effectiveness research [6,7]. This movement calls for greater participation of private practice environments in clinical research [7].

One of the reasons often given for the meager benefits that have been found with various LBP treatments is that these treatments are generally applied generically, without regard for specific characteristics of each patient, whereas the LBP population is a heterogeneous group, requiring individualized care [8]. Developing a strategy by which treatments can be targeted to the specific needs of patients has been identified as a research priority [9,10].

In recent years there has been a movement away from the biomedical model for understanding the LBP experience toward a biopsychosocial model [11-15]. That is, LBP has increasingly been recognized as involving somatic, neurophysiological and psychological factors that all contribute to the clinical picture clinicians encounter. In addition, it has been recognized in recent years that, while there are several individual treatments for LBP that have evidence of effectiveness, the effects sizes of these treatments are generally small [4]. It was been argued that this is likely because patients with LBP have individual needs and taking an approach that identifies the key features in each case, so that treatment can be tailored to those key features, provides the greatest benefit to the patient [16]. However little information is available on the relative efficacy of any particular systematic approach to applying the biopsychosocial model in clinical practice.

A diagnosis-based clinical decision guide (DBCDG) has been proposed for the purpose of guiding clinicians in applying biopsychosocial concepts to the diagnosis and management of patients with LBP [16]. This has been referred to in previous publications as a diagnosis-based clinical decision rule. The approach evolved from the evidence regarding the somatic, neurophysiological and psychological factors that have been found to contribute to suffering in patients with LBP, along with those treatments that have been found to be effective in patients with LBP [17]. It attempts to respond to the challenge of applying the biopsychosocial model and providing individualized treatment programs based on the particular features of each patient.

Cohort studies documenting the outcome of treatment of subsets of LBP patients have been published and the results appear promising [18-20]. However, more research is needed to determine the generalizability of these findings as well as whether they can be replicated in controlled studies. The primary purpose of this study is to document the types of working diagnoses in patients with LBP that are formed by clinicians trained in the use of the DBCDG. This will serve as the basis for further refining the approach in an attempt to improve diagnostic accuracy.

## Methods

The study protocol was approved by the Institutional Review Board of New York Chiropractic College (protocol #09-04). It was also reviewed by the Health Insurance Portability and Accountability Act (HIPAA) compliance officer of the facility at which the data were gathered and was deemed to be in compliance with HIPAA regulations. All subjects signed informed consent forms, agreeing to have their data included in the study.

Data were gathered prospectively in consecutive patients seen at the Rhode Island Spine Center between 2/7/08 and 2/26/09.

### Participants

Patients were included in the study if they 1) had LBP (defined as pain between the thoracolumbar junction and the buttocks, with or without lower extremity pain; 2) were age 18 years or older; 3) provided informed consent; 4) were able to communicate well in English; 5) had a Bournemouth Disability Questionnaire (BDQ) score of 15 or higher.

### Clinical Examination

All examinations were carried out by one of two chiropractic physicians, one with over 20 years experience and the other with over 9 years experience, or by a physical therapist with over 10 years experience. All had a minimum of 50 hours of postgraduate training in the McKenzie method. The physical therapist also had 80 hours of postgraduate training in manual therapy. Several discussions between the examiners took place over the course of five years prior to commencing data gathering on the application of the DBCDG. This occurred in the form of monthly clinical meetings in which the application of the DBCDG in particular patients was discussed as well as recent developments in the literature related to the evaluation and management of patients with LBP. History and examination were performed according to the usual course of patient care at the Rhode Island Spine Center.

Details of the DBCDG are published elsewhere [16,17] but the approach is based on three questions of diagnosis:

1. Are the symptoms with which the patient is presenting reflective of a visceral disorder or a serious or potentially life-threatening disease?

The purpose of this question is to identify signs and symptoms suggestive of non-musculoskeletal problems for which LBP may be among the initial symptoms. Gastrointestinal and genitourinary disorders are included in addition to such "red flag" disorders as infection and malignancy.

2. From where is the patient's pain arising?

With this question the clinician investigates distinguishable characteristics of the pain that may allow treatment decisions to be made. In most cases, the exact tissue of origin cannot be unequivocally determined, however several studies have found that patients can be distinguished based on historical and examination characteristics [21-27] and treatment decisions can be made based on these characteristics [28].

3. What has gone wrong with this person as a whole that would cause the pain experience to develop and persist?

With this question the clinician attempts to identify factors that may serve to perpetuate the ongoing pain experience. These factors may involve somatic, neurophysiologic or psychological processes [16].

Following each new patient encounter the answers to the three questions of diagnosis were documented on a standardized form (see Additional file 1). These data, along with patient demographic data and data from standardized outcome measurement instruments were then entered on a spreadsheet by a chiropractic intern.

**Figure 1:**
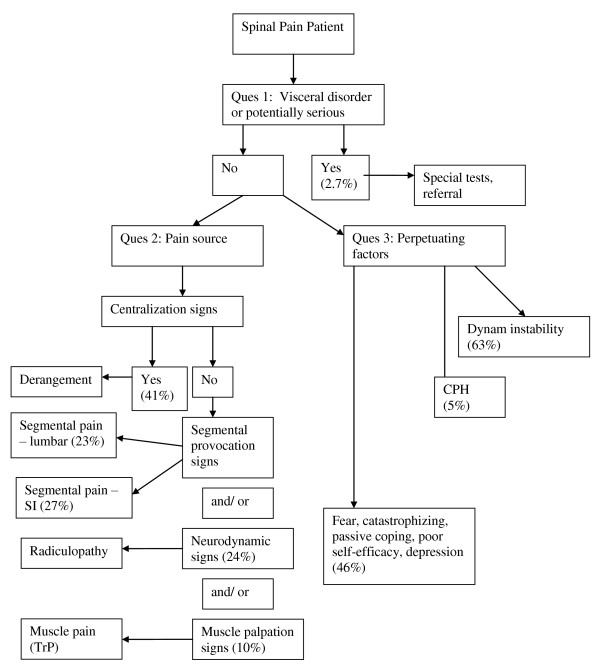
**Diagnostic algorithm for the application of the DBCDG**. Reprinted with permission from: Murphy DR, Hurwitz EL. A theoretical model for the development of a diagnosis-based clinical decision guide for the management of patients with spinal pain. BMC Musculoskelet Disord 2007;8:75. cerv = cervical; thor = thoracic; lumb = lumbar; SI = sacroiliac; TrP = trigger point; CPH = central pain hypersensitivity; dysfx = dysfunction; catastroph = catastrophizing.

**Figure 2:**
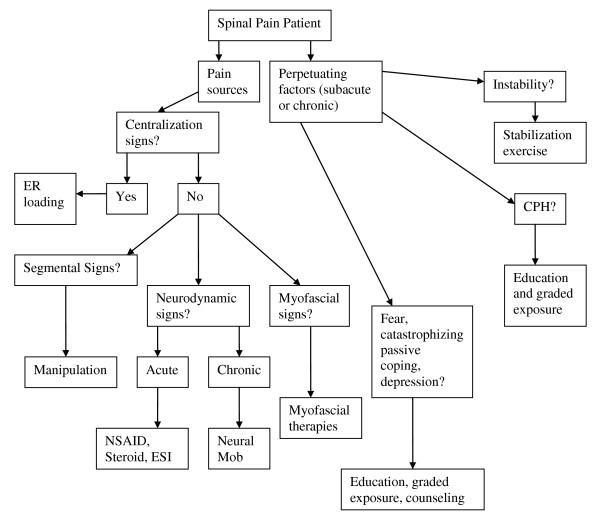
**Management algorithm for the application of the DBCDG**. Reprinted with permission from: Murphy DR, Hurwitz EL. A theoretical model for the development of a diagnosis-based clinical decision guide for the management of patients with spinal pain. BMC Musculoskelet Disord 2007;8:75. ER = end range; NSAID = non-steroidal anti-inflammatory drugs; ESI = epidural steroid injection; mob = mobilization; CPH = central pain hypersensitivity.

The answers to the three questions of diagnosis allows for the development of a working diagnosis (Figure 1) upon which a trial of treatment can be based (Figure 2). The working diagnosis is often multifactorial and may include a combination of biological and psychological processes as well as the social context in which these occur.

In seeking an answer to the first question of diagnosis (rule out visceral or serious disease) standard history and examination procedures were used. In cases in which it was warranted, special tests such as radiographs, MRI or blood tests were ordered.

In seeking answers to the second question of diagnosis (source of the pain), four signs were considered [16,17]:

1. Centralization signs, detected through historical factors that are associated with disc pain [23] and by using the end-range loading examination procedure of McKenzie [29].

2. Segmental pain provocation signs, detected through historical factors that are associated with lumbar facet or sacroiliac pain [23] and through the pain provocation tests of Laslett, et al [22,23,25,30]. Evidence suggest that centralization signs must be ruled out prior to consideration of segmental pain provocation signs [22,30]. Therefore, segmental pain provocation signs were only considered relevant if centralization signs were absent.

3. Neurodynamic signs, detected through historical factors associated with radiculopathy and neurodynamic tests designed to provoke nerve root pain [31-34].

4. Myofascial signs, detected through palpation of myofascial tissues [35]. These signs were only considered relevant if the clinician felt they were separate and distinct from the other signs.

In seeking answers to the third question of diagnosis (perpetuating factors), three factors were considered [16]:

1. Dynamic instability, detected through clinical tests of motor control for the lumbopelvic spine [36-43].

2. Central pain hypersensitivity, detected through observation of pain behavior in response to stimuli as well as through Waddell's nonorganic signs [44]. A threshold of 3/5 nonorganic signs was used as this is the threshold that has been used in previous studies as being significant for the presence of non-organic pain behavior [45].

3. Psychological factors. Fear beliefs were measured using the 11-item Tampa Scale for Kinesiophobia (TSK) [46]. A score of 27 was considered indicative of clinically meaningful fear beliefs. This number was adapted from Vlaeyen, et al [47] who used a cutoff score 40 using a previous 17-item version of the TSK and Woby (personal communication 3 August, 2009) whose unpublished data suggested a score of 26 to 27 to be associated with clinically meaningful fear beliefs. In addition, two questions from the Coping Strategies Questionnaire [48] which have previously been found to be predictive of changes in disability in LBP patients [49] were used to measure patients' perception of their control over the pain. At the time this study was conducted no data were available regarding whether a particular score with these questions constitutes a threshold for clinically meaningful difficulty with coping strategies. The depression subscale of the BDQ [50] was used to measure depression. As with the coping strategies questions, no data were available at the time of the study by which to determine a threshold for clinical significance with this question.

Each patient completed the full BDQ [50] and the total score from this questionnaire score was recorded. The initial subscale of the BDQ consists of a Numerical Rating Scale for pain intensity (NRS) [51], a scale in which the patient is asked to rate the average intensity of the pain over the past week on a 0-10 scale with "0" representing "no pain" and "10" representing "worst possible pain". This score was also recorded.

### Treatments

Treatment was left to the discretion of the primary treating clinician based on the diagnosis, and in general a "team approach" was taken. In the context of the DBCDG, these are the treatments that were applied:

In response to the findings or the second question of diagnosis (source of the pain):

Centralization signs: End range loading maneuvers in the direction that produced centralization [29]. Because centralization signs are believed to reflect disc pain [21], distraction manipulation [52] was also used, as this has been found to decrease intradiscal pressure [53] and has been shown to be helpful in patients with LBP in general [54].

Segmental pain provocation signs: As joint manipulation has been shown to have both neurological [55] and biomechanical [56] segmental effects and has been found to be beneficial in patients with LBP in general [57], this was applied as the treatment of choice in patients with segmental pain provocation signs.

Neurodynamic signs: In the acute stage, anti-inflammatory measures were pursued via referral. This was in the form of non-steroidal anti-inflammatory medications, oral steroids or epidural steroid injections [58], depending on the diagnosis. In the subacute or chronic stage, neural mobilization was used [59].

Myofascial signs: Myofascial therapies such as ischemic compression and post-isometric relaxation [60] were used if the myofascial signs were deemed clinically relevant by the treating clinician.

In response to the third question of diagnosis (perpetuating factors):

Dynamic instability: Patients diagnosed with dynamic instability were treated with stabilization exercise [61,62].

Central pain hypersensitivity: Education was provided regarding the nature of pain for the purpose of helping the patient understand that the intensity of pain was not related to extensive "tissue damage" [63,64]. In addition, graded exposure [65] was applied in which patients were exposed to movements, positions and activities that provoked their pain to a level they could handle and the stimulus was continued until habituation occurred [66]. Graded exposure was only applied in the subacute or chronic stage, not in acute patients.

Fear, catastrophizing, passive coping, depression, poor self-efficacy: Education was provided for the purpose of correcting misperceptions regarding the nature of pain [63]. In addition, graded exposure was applied [67]. Occasionally patients were referred for cognitive-behavioral therapy [68].

The treatment algorithm can be found in Figure 2.

### Statistical analysis

Descriptive statistics were used to characterize the study population. Frequencies, percentages, and 95% confidence intervals were computed for categorical variables; means, standard deviations, medians, and ranges were computed for continuous variables. Data management and statistical analyses were conducted with Microsoft Excel and SAS (version 9.1, Cary, NC).

## Results

Data were gathered on 264 patients, 63% of whom were female. The mean BDQ score was 40 and the mean pain intensity was 7/10. Baseline characteristics are presented in Table 1.

**Table 1:** Baseline characteristics

Variable	Mean (SD)	Median (IQR)	Range
Age (years)	49.0 (16.2)	48.0 (24)	18-86

Duration (days)	912.8 (2639.4)	106.0 (337)	1 day - 54 years

BDQ	40.5 (13.4)	39.0 (20)	14-70

NRS	6.9 (1.9)	7.0 (2)	2-10

Tampa	25.1 (6.1)	25.0 (8)	11-42

Coping	5.6 (2.5)	6.0 (3)	0-12

Depression	4.3 (3.2)	5.0 (6)	0-10

SD = Standard deviation; IQR = Interquartile range; BDQ = Bournemouth Disability Questionnaire; NRS = Numerical Rating Scale (pain); Tampa = Tampa Scale for Kinesiophobia

Regarding the first question of diagnosis (rule out visceral or serious disease), 2.7% of patients were positive. Data regarding the second (source of the pain) and third (perpetuating factors) questions of diagnosis are provided in tables 2 and 3, respectively. The most common sign under the second question of diagnosis was centralization (41.1%) followed by sacroiliac segmental pain provocation signs (27.0%). The most common sign under the third question of diagnosis was dynamic instability (63.3%) followed by fear (39.8%).

**Table 2:** Responses to the second question of diagnosis.

Diagnostic sign	Percent (95% CI)
Centralization sign	41.1 (35.1 - 47.0)

Segmental pain provocation sign (lumbar)	23.3 (18.2 - 28.4)

Segmental pain provocation sign (sacroiliac)	27.0 (21.6 - 32.4)

Neurodynamic sign	23.9 (18.7 - 29.0)

Myofascial sign	10.3 (6.6 - 13.9)

CI = confidence interval

**Table 3:** Responses to the third question of diagnosis

Diagnostic sign	Percent (95% CI)
Dynamic instability (lumbar)	46.6% (95% CI 40.6 - 52.6)

Dynamic instability (pelvic)	16.7 (12.2 - 21.2)

Central pain hypersensitivity	5.3 (2.6 - 8.0)

Fear	39.8 (33.9 - 45.7)

Passive coping	3.0 (1.0 - 5.1)

Depression	3.0 (1.0 - 5.1)

CI = confidence interval

## Discussion

In recent years, spending on the diagnosis and management of patients with LBP has dramatically increased, yet this has not resulted in improved outcomes in terms of patient suffering and disability rates [4]. As such, there is a great need for improved decision making in the care of patients with LBP. Specifically, there is a need to identify characteristics of each individual's condition that allow clinicians to make treatment decisions. In addition there is a great need for research that documents the clinical processes and outcomes that occur in the "real-world" environment of clinical practice as a contributor to comparative effectiveness research [6,7]. This study was part of a broad research strategy to respond to the need for practice-based research by investigating and refining the clinical utility of the DBCDG for patients with LBP. The purpose was to document the types of diagnostic features identified and the frequency of the clinical findings.

Centralization signs were found in 41% of patients. This is similar but slightly lower than the 45-50% prevalence of this sign found in other studies of patients with LBP [21,69,70]. It is substantially lower than the 61.5% prevalence found by Murphy, et al [20] in a population of patients with radiculopathy secondary to herniated disc. In the present study data were only gathered at the initial visit. It has been found that when the determination of the centralization response occurs over the course of several visits, the process is more accurate [71]. Thus, the percentage of patients who were centralizers may be underestimated in the present study.

The prevalence of segmental signs involving the SI joint was 27%. This is similar to the 31% reported by DePalma, et al [72] but substantially higher than the 13% reported by Maigne, et al based on diagnostic injections [73]. This is interesting in that the means of identifying these signs have been found to have high sensitivity and specificity when using injection as a Gold Standard [23,25]. However, these validity studies used single, rather than double, joint blocks. The prevalence of 23% for segmental signs related to the facet joints was within the range of 15-40% reported previously [74] and very similar to the 18% reported by DePalma, et al [72]. The prevalence of the diagnosis of muscle palpation signs was low (10%). No prevalence data on myofascial pain is found in the literature, but it is the perception of the clinicians involved in this study, based on discussions over the five years prior to the gathering of these data, that muscle palpation signs are very common but often do not require specific treatment, and that applying treatment based on these signs does not positively impact outcome. This may explain why these signs were deemed clinically relevant in only a small percentage of patients. Further research is needed to investigate this perception. The relatively low prevalence of muscle palpation signs may also reflect the fact that the reliability of palpation to identify myofascial trigger points in the lumbar spine is relatively low [75-77].

There were three factors under the third question of diagnosis (perpetuating factors) for which the prevalence was quite low. Only 5% of patients were identified to have central pain hypersensitivity and only 3% were identified to have each of passive coping and depression. As these factors have been found to be significant in the development of chronic LBP [78-80], it is likely that the low prevalence of the diagnosis of these factors in this study represents under-recognition. However, as the mean duration of pain was only 109 days, it may be that the prevalence would naturally be higher in a cohort of patients with more long-standing pain. Another possibility is that this cohort did not display these features or that a sampling error led to low prevalence. It also may be that the means used in this study to identify these factors were suboptimal. In the case of central pain hypersensitivity, there is no well-established means of identification. Utilizing Waddell's non-organic signs with a threshold of a score of 3/5 may be of insufficient sensitivity to be used as a screening tool for central pain hypersensitivity. In addition, there may be other methods, such as pressure algometry [81], that may be useful in the detection of central pain hypersensitivity. Criteria have been developed by Smart, et al using a Delphi process [82], three factors of which have been found to have discriminative validity for the identification of central pain [83]. This may be a more useful approach than the one taken here and further research is required to investigate this. In the case of passive coping and depression, the scales used to identify these factors had no established threshold score that identifies the presence of clinically meaningful problematic coping strategies and depression. The mean score on the coping strategies questions was 5.6 out of a possible 12 and on the depression subscale on the BDQ was 4.3 out of a possible 10. A recent study found that a baseline coping score of less than 8 had the highest sensitivity and score of less than 4 had the greatest specificity in identifying a LBP patient who is not likely to experience clinically meaningful improvement in pain and disability [84]. These data will be used as the basis for further investigation that attempts to establish thresholds for clinical meaningful coping problems. It is expected that this knowledge will increase the validity of these questions when attempting to identify patients with problematic coping

strategies and depression. Other important psychological factors that are of importance in patients with LBP, such as catastrophizing [85], poor self-efficacy [85], hypervigilance for symptoms [86] and cognitive fusion [87] were not specifically measured. There is some evidence that the various psychological factors interact, rather than occurring in isolation [88-91] and that identification of more than one factor, but not necessarily all factors is adequate [92]. As this was a practice-based research project that is part of the investigation of identification of key elements in the perpetuation of LBP in a "real-world" environment, it was decided that fear, coping and depression would be measured rather than attempting to measure all potentially relevant factors. Further work is needed to determine whether this is a worthwhile approach for clinicians.

This study had several limitations. First, the sample size was only 264 patients. A larger sample would have increased the study's scientific rigor. In addition, all data were gathered at a single clinic and thus it is not known whether the information is generalizable. Also the design was observational and the practitioners were not blinded to the findings on each patient. Finally, because this was a pragmatic study in which data were gathered during the normal course of clinical care detailed information regarding psychological factors was not obtained as this would have required patients to fill out several questionnaires. On the other hand, the fact that this study was carried out in a real-world environment may be a strength, in that it suggests that the information applies to the environment in which patients are most commonly cared for as opposed to the controlled environment of a research center.

Future studies will seek to determine correlations and patterns among the various diagnostic factors, the utility of the coping strategies and depression questions that were used, the inter-examiner reliability of the diagnostic strategy, and ultimately efficacy of the approach. Preliminary data suggests that outcomes in select patients groups may be favorable [18-20,93], but this is based on observational studies without randomization or control.

## Conclusion

The DBCDG can be applied in a private practice setting. It appears that patients with LBP can be distinguished on the basis of the findings of this approach, and treatment plans can be formulated based on the diagnosis by utilizing this strategy. Future research is needed to investigate the validity of the questions used in this study to identify problematic coping strategies and depression and to seek improved means of identifying central pain hypersensitivity. Further research is also needed to investigate correlations between the diagnostic findings, reliability of the diagnoses and efficacy of treatment based on the DBCDG.

## Competing interests

The authors declare that they have no competing interests.

## Authors' contributions

DRM originally conceived of the study and served as an examiner. He was also the main writer of the manuscript. ELH was responsible for statistical analysis and writing and editing the manuscript. Both authors read and approved the final manuscript.

## Supplementary Material

Additional file 1 **Standardized form on which the answers to the three questions of diagnosis were documented**. Click here for file
